# *Cachrys* spp. from Southern Italy: Phytochemical Characterization and JAK/STAT Signaling Pathway Inhibition

**DOI:** 10.3390/plants11212913

**Published:** 2022-10-29

**Authors:** Maria Rosaria Perri, Michele Pellegrino, Stefano Aquaro, Fabiola Cavaliere, Carmine Lupia, Dimitar Uzunov, Mariangela Marrelli, Filomena Conforti, Giancarlo Statti

**Affiliations:** 1 Department of Pharmacy, Health and Nutritional Sciences, University of Calabria, 87036 Rende, Italy; 2 Mediterranean Etnobotanical Conservatory, 88054 Sersale, Italy; 3 National Etnobotanical Conservatory, 85040 Castelluccio Superiore, Italy; 4 National Museum of Natural History, Bulgarian Academy of Sciences, 1000 Sofia, Bulgaria

**Keywords:** Apiaceae, bergapten, *Cachrys ferulacea*, *Cachrys libanotis*, *Cachrys pungens*, furanocoumarins, JAK/STAT, IL-6, inflammation, TNF-α

## Abstract

Different phytochemical compounds have been demonstrated to modulate the JAK/STAT signaling pathway. Here, three *Cachrys* species from Southern Italy were investigated for both the phytochemical profile and the potential anti-inflammatory properties. The aerial parts were extracted with methanol through Naviglio Extractor^®^, an innovative solid-liquid extraction technique that allows to obtain high quality extracts by working with gradient pressure. Extracts were analyzed with GC-MS and standardized in furanocoumarin content, resulting rich in xanthotoxin, bergapten and isopimpinellin. Given the known ability of bergapten to inhibit the JAK/STAT signaling pathway by decreasing the levels of pro-inflammatory cytokines (TNF-α, IL-6) and inflammatory mediators (NO) in RAW 264.7 cells activated by LPS, *Cachrys* extracts were investigated for their biological properties. The results obtained in this study showed that *Cachrys pungens* extract, presenting the highest content in furanocoumarins (7.48 ± 0.48 and 2.94 ± 0.16 mg/50 mg of extract for xanthotoxin and bergapten, respectively), significantly decreased STAT3 protein levels, pro-inflammatory cytokines (TNF-α, IL-6) and increased IL-10 anti-inflammatory cytokine. *Cachrys ferulacea* significantly decreased JAK2 phosphorylation, being even more effective than bergapten. In conclusion, investigated extracts could be potential candidates for the search of novel anti-inflammatory agents acting via inhibiting the JAK/STAT signaling pathway.

## 1. Introduction

Nature has always provided a wide range of bioactive compounds with extraordinary properties and endless application: it can be considered the origin of pharmacology and therapeutics, definitively. In fact, the biochemical pathways that take place inside plants lead to the production of secondary metabolites which show a wide range of not negligible therapeutics activities [1]. Unlike primary metabolites, these are organic compounds not responsible for growth, development and reproductive functions of the plant, and are often a mixture of structurally similar chemical molecules [2]. For many years, nature was used as a source of pure single molecules isolated by plants in drug discovery field; nowadays, herbal medicinal products find their own application both in traditional and complementary medicine. Moreover, the World Health Organization (WHO) created the Traditional Medicine Strategy (2014–2023), that involves herbal medicine as medicinal therapies [1]. 

Secondary plant metabolites are the result of biological evolution under certain ecological conditions: when these molecules interact with each other, they can result in independent, additive, synergetic or antagonistic effects. An additive activity corresponds to the sum of each component effect, while a synergic activity means a super-additive effect during the combined action. The antagonistic effect, instead, translates itself in a lower activity than the own single effect [3]. 

There is a growing interest in chemoprevention by utilizing natural compounds such as phytochemicals, minerals and vitamins. Evidences show that phytochemicals can affect proliferation, differentiation, apoptosis, evasion and angiogenesis. It is recognized that the aberrant activation of the JAK/STAT signaling pathway is involved in tumorigenesis and it can promote the transformation process of preneoplastic lesion into malignant tumor. More than 50 cytokines, secreted glycoproteins able to act as intercellular messengers, are involved in the final activation of the JAK/STAT signaling pathway. They play a key role by binding specific membrane receptors on target cells containing intracellular domains linked to JAK family members. The cytokine binding leads to JAK autoactivation: in this way, phosphorylated JAK acts as docking sites for STATs proteins. At this level, JAK phosphorylates STAT, that can translocate into the nucleus in order to activate genes inducing proliferation, differentiation, growth and apoptosis of target cells [4]. 

Bergapten (5-methoxypsoralen) is a naturally occurring coumarin known for its photosensitizing activity [5]. Evidences show that this molecule exerts several pharmacological activities such as neuroprotection, organ protection, anticancer, anti-inflammatory, antimicrobial and antidiabetic effects [6]. Moreover, studies showed that bergapten has neuropharmacological effects in Alzheimer’s disease, depression and anticonvulsant activity. The bergapten potential in the treatment and prevention of cancer was recognized in liver, breast, lung, colorectal cancer and melanoma through apoptosis and cell proliferation inhibition mechanisms [7]. 

The anti-inflammatory activity of bergapten is noteworthy: a recent study by Zhou and coworkers [8] showed how this molecule inhibited the production of TNF-α, IL-1β, IL-6, PGE2 and NO, besides the suppression of iNOS and COX-2 expression in RAW 264.7 murine macrophage cells, previously stimulated with LPS. As a result, the suppression of the JAK/STAT signaling pathway and the reduced production of ROS was observed.

Bergapten is widely spread in *Citrus* fruit essential oils, in particular in bergamot essential oil, and in Apiaceae, Rutaceaea and Fabaceae plant families [9]. 

*Cachrys* genus, belonging to the Apiaceae family, consists of about 100 species spread in all the Mediterranen area. Three species, *C. libanotis* L., *C. ferulacea* (L.) Calest. and *C. pungens* Jan, in particular, grow in Southern Italy (Figure S1). The name *C. ferulacea* is a synonymous of *Prangos ferulacea* Lindl., being the *Cachrys* group divided into a number of genera including not only *Cachrys* and *Prangos*, but also *Alocacarpum*, *Azilia*, *Bilacunaria*, *Diplotaenia*, *Eriocycla* and *Ferulago* [10]. The major secondary metabolites of these species were identified in coumarins and furanocoumarins, among which bergapten represents one of the most interesting molecules [11]. 

The activity related to furanocoumarins structure depends on the furan ring and the coumarin linear or angular backbone [12]. Following the “similar property principles”, structurally similar molecules should have similar properties. In fact, one of the central premises of medicinal chemistry is that similar molecules exert the same biological activity, as tradition of drugs demonstrated: for example, β-lactams, usually have anti-bacterial activity, or aromatic nitro compound are known to be a mutagenic class of compounds [13,14]. 

At the state of the art, a potential activity of bergapten in inhibiting the JAK/STAT signaling pathway was showed [8]: starting from this data, the experimental design provides to compare bergapten as a pure single molecule to *Cachrys* standardized extracts. 

*Cachrys* extracts were obtained with an innovative technology for solid-liquid extraction based on the use of the “Naviglio Extractor^®^”. The extraction method is based on Naviglio’s principle, the idea that by generating a negative pressure gradient between the inner and the outside of the matrix, it is possible to achieve extracts. This method changed the common theory that solid-liquid extraction can be obtained only by diffusion and osmosis method. Moreover, the extraction with Naviglio extractor^®^ doesn’t need heat in the system, it works with gradient pressure only: this is a not negligible advantage because it allows to extract thermolabile substances from matrix, and this results in a better quality of extracts. An extractive cycle consists in a static and a dynamic phase: the first phase works on 9 atm, the liquid penetrates the matrix, while in the second phase the pressure is quickly removed and carried to the atmospheric pressure. This imbalance is important because it allows the liquid to be enriched with bioactive substances in a very short time and without the help of heat [15,16]. 

In a previous study, we already reported the interesting coumarin content of *C. libanotis* extract [11]. Here, the phytochemical characterization and quantitative analyses were performed by means of gas chromatography—mass spectrometry (GC-MS) on the other two *Cachrys* species, and Principal Component Analysis (PCA) was used to highlight the differences among the composition of the three extracts.

Obtained extracts were then tested for their in vitro anti-inflammatory potential by evaluating the ability to affect the LPS-stimulated release of pro-inflammatory (TNF-α and IL-6), anti-inflammatory cytokines (IL-10) and NO mediator in RAW 264.7 cells. Moreover, the modulation of the JAK/STAT signaling pathway was evaluated.

## 2. Results and Discussion

### 2.1. Phytochemical Profile

The plant material (aerial parts) of the three *Cachrys* species was extracted through pressurized cyclic solid-liquid extraction by means of the Naviglio Extractor^®^. The highest extraction yield was estimated in *C. libanotis* extract (12.6%, Table 1). 

Samples were evaluated for their total phenolic and flavonoid content: *C. libanotis* sample showed the highest content of polyphenols, with a value of 12.80 ± 0.10 mg/g of dry plant material, followed by *C. pungens* and *C. ferulacea* extracts, with data equal to 8.64 ± 0.42 and 4.12 ± 0.24 mg/g of dry plant material, respectively (Table 1). This trend was not confirmed by the evaluation of total flavonoid content in which *C. pungens* extract resulted to be the best sample (0.32 ± 0.01 mg/g), followed by *C. ferulacea* and *C. libanotis* extracts.

It has been clearly demonstrated that the amount and composition of phenolic constituents may vary depending on different factors. Both climatic and agrotechnical conditions in cultivation and harvesting, the collection time, the storage conditions, and genetic factors are important [17].

The interspecies differences among plants belonging to the same genus are intrinsic factors that mostly contribute to the interspecies variability of the phytochemical composition [18]. Different studies underlined a varieties-dependent variability in the phenolic content. For instance, Boneza and colleagues highlighted that both the selection of cultivar and seed origin were important factors able to affect phenolic compounds concentration and the resulting antioxidant properties in *Melissa officinalis* varieties [19]. Clear differences were also observed, for example, in the phenolic profiles of different *Amaranthus* species [20], *Salvia* spp. [21], and *Thymus* spp. [22]. Bazdar and coworkers investigated the differences occurring in the phenolic and flavonoid composition of extracts from different plant parts, flowers and leaves, of *Prangos ferulacea* (Syn. *C. ferulacea*). Some significant differences in the antioxidant as well in the chemical composition were verified [23]. 

The phytochemical composition of the three *Cachrys* species was assessed with GC-MS. In our previous works, we already assessed the interesting furanocoumarins content of some *Cachrys* extracts, among which *C. libanotis*, which was tested for its photocytotoxic properties in combination with the UVA radiations on melanoma cancer cells [11,24]. Here, the phytochemical profiles of three *Cachrys* spp. were compared. *C. pungens* showed the highest content both in furanocoumarins and coumarins, while *C. libanotis* presented the only identified pyranocoumarin.

**Figure 1:**
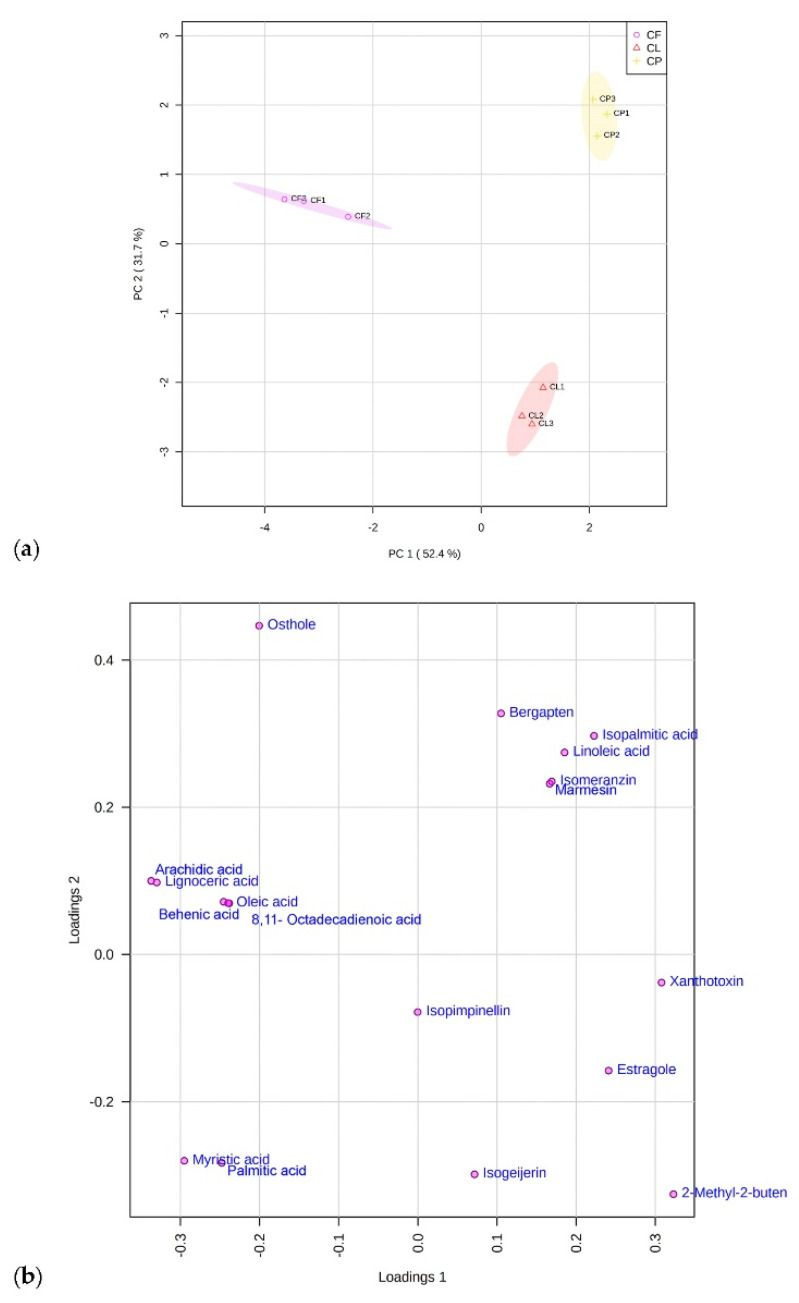
Scores (**a**) and loading plots (**b**) generated using the first two principal components (PC1 vs. PC2).

Principal Component Analysis (PCA) allowed exploring the data patterns and obtaining a clear overview of the distribution of identified metabolites in the three extracts. Figure 1 describes the scores (a) and loadings plots (b) by using the first and the second principal components (PC-1 vs. PC-2), with a total explained variance of 84.1%. The *C. pungens* (CP) samples, located in the top right half of the scores plot, were characterized by the highest content of furanocoumarins compared to the other samples. 

**Figure 2:**
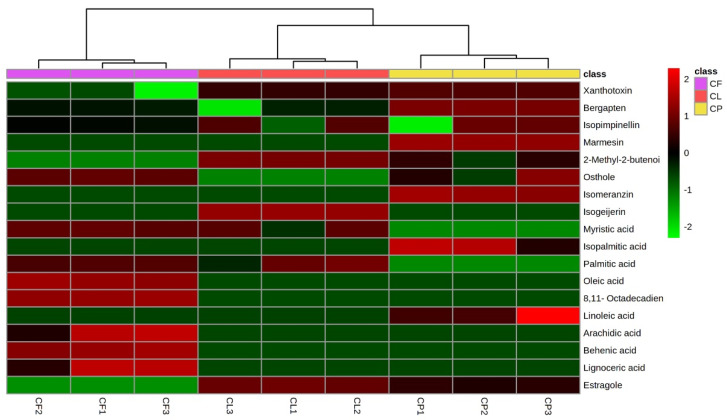
Heat map of identified phytochemicals in the three *Cachrys* species. CF: *C. ferulacea*; CL: *C. libanotis*; CP: *C. pungens*.

Figure 2 reports the heat map in which the differences in the relative content of significant discriminant secondary metabolites are visualized.

**Figure 3:**
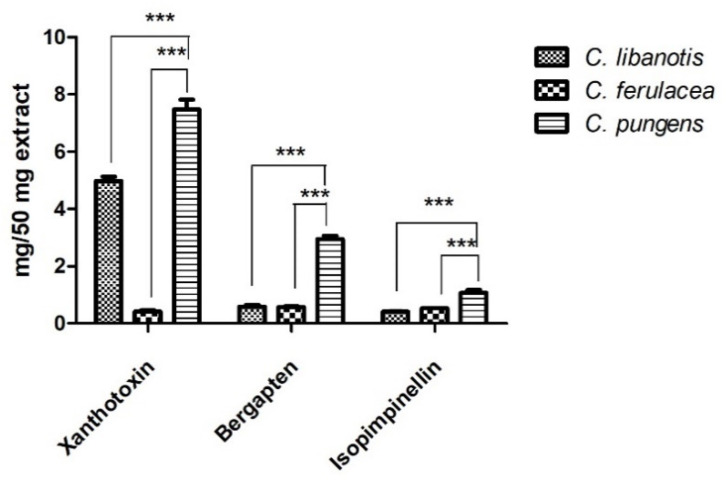
Graphical representation of quantitative GC-MS analysis. Data are expressed as mean ± S.D. (n = 3). Statistical differences *** *p* < 0.001 (Students’ *t* test).

The phytochemical profile of the extracts was completed with a quantitative GC-MS analysis aimed at evaluating the amount of three furanocoumarins by using the external standard method. Among the three investigated molecules, xanthotoxin seemed to be the one present in higher quantity both in *C. libanotis* and *C. pungens* extracts, with amounts of 4.98 ± 0.21 and 7.48 ± 0.48 mg/50 mg extract, respectively (Table 2, Figure 3). 

The highest content of bergapten was assessed in *C. pungens* extract (2.94 ± 0.16 mg/50 mg extract), while comparable values were detected in *C. libanotis* and *C. ferulacea* with results equal to 0.59 ± 0.08 and 0.57 ± 0.04 mg/50 mg of extracts, respectively. Isopimpinellin was found mostly in *C. pungens* sample (1.07 ± 0.14 mg/50 mg of extract), while the lowest quantity was detected in *C. ferulacea*. 

*C. pungens* extract presented the highest content of furanocoumarins, being xanthotoxin the most abundant one. The only one sample in which the content of bergapten exceeds that of xanthotoxin was *C. ferulacea* (Figure 3).

### 2.2. Phytochemical Profile Effects of Bergapten and Cachrys Extracts on LPS-Induced Production of Pro-Inflammatory, Anti-Inflammatory Cytokines and Mediators (NO) in RAW 264.7 Cells

Since the production of pro-inflammatory cytokines such as TNF-α, IL-6 and mediators like nitric oxide (NO) play a central role in inflammatory processes, macrophage cells activated with LPS were used in order to evaluate the anti-inflammatory potential of samples under investigation. 

The effect of bergapten and *Cachrys* extracts against pro-inflammatory (TNF-α and IL-6) and anti-inflammatory (IL-10) cytokines was evaluated by means of ELISA test. RAW 264.7 cells were pretreated for 30 min and then stimulated with LPS for 24 h. When time runned out, surnatant was collected and ELISA assay started. Bergapten, at a final concentration of 10 µM was used as reference compound (positive control) and compared with *Cachrys* extracts, previously prepared in such a way that sum of three furanocoumarins, identified and quantified by GC-MS analyses, corresponded to the dose of bergapten 10 µM (2.16 µg/mL, sample) and half of it, 5 µM (1.08 µg/mL, sample b). The aim of this study was to compare the activity of the single active principle (bergapten), with activity of extracts containing not only three furanocoumarins, but also a huge quantity of other compounds unknown and unquantifiable. Utilized formulations are reported in Table 3.

Macrophages stimulated with LPS are considered the largest source of release of pro-inflammatory cytokines. Previous literature studies showed, in fact, that the production of TNF-α and IL-6 resulted significantly increased after LPS activation, while in normal condition, their levels result to be basal [25]. 

**Figure 4:**
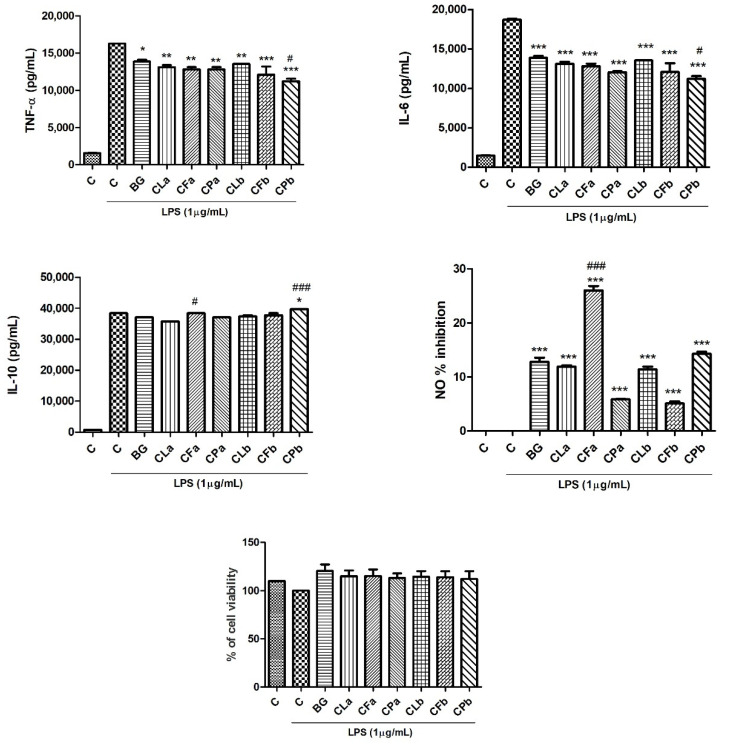
Bergapten (BG) was used as positive control. CL: *C. libanotis*; CF: *C. ferulacea*; CP: *C. pungens*. Formulations “a” and “b” were prepared as reported in the Materials and Methods Section 3.2. Significant difference versus control with LPS: * *p* < 0.05; ** *p* < 0.01; *** *p* < 0.001; significant difference versus bergapten (positive control): # *p* < 0.05; ### *p* < 0.001 (Dunnett’s multiple comparison test).

As shown in Figure 4, bergapten is able to decrease the release of the pro-inflammatory cytokines (TNF-α and IL-6) if compared to control. 

For what concerns TNF-α reduction, all tested extracts, both at highest and lowest concentrations, were effective. CPb exerted the best inhibitory activity, as it was even more effective than bergapten, used as reference compound (*p* < 0.05, Dunnett’s multiple comparison test). The same trend was observed regarding IL-6: all the samples significantly reduced the cytokine release, with CPb showing the best inhibitory activity. According to these data, Bose and coworkers demonstrated that bergapten induced a significant dose-dependent inhibition of TNF-α and IL-6 in peripheral blood mononuclear cells (PBMCs) stimulated with LPS [26]. Singh and coworkers demonstrated that bergapten decreased levels of TNF-α and IL-6 in plasma of animals treated with formalin, acetic acid and carrageenan in order to induce neurogenic and inflammatory hyperalgesia and inflammation [27].

Bergapten and *Cachrys* extracts were also screened for their effects on the release of IL-10 anti-inflammatory cytokine. *Cachrys pungens* (CPb) was confirmed to be the most effective extract, even more effective than bergapten, used as reference compound. As Zhou and coworkers demonstrated, bergapten reduced the release of TNF-α, IL-6 and NO and improved the production of IL-10 in a dose-dependent manner [8]. 

Moreover, the inhibition of NO oxidized products was assessed by using Griess reagent. In this case, CFa showed the highest inhibitory effect, with a percentage higher than 20%. Yang and coworkers showed, for the first time, that bergapten is able to reduce NO level in tail-cutting-induced inflammation in zebrafish model [28]. Wang and coworkers screened a series of furanocoumarins on NO production in LPS stimulated RAW 264.7 cells: results showed that xanthotoxin is a potential NO inhibitor with an IC_50_ value of 16.6 µg/mL [29]. 

Finally, to exclude the possibility that the inhibition of pro-inflammatory cytokines and mediators and the increase of IL-10 was due to cytotoxicity, a SRB test was performed. This assay showed that all the tested formulations did not affect cell viability (Figure 4). This demonstrated that samples could inhibit the release of pro-inflammatory cytokines and mediators such as TNF-α, IL-6 and NO in RAW264.7 cells subjected to LPS stimulation in non-cytotoxic doses.

### 2.3. Effects of Bergapten and Cachrys Extracts on LPS-Induced JAK/STAT Signaling Pathway Activation

The JAK/STAT signaling pathway plays a key role in the inflammatory process: it results close to growth, survival, proliferation, metastasis and angiogenesis in many different cancer cell lines, so it can actually be considered a potential therapeutic target in cancer research field [30]. STAT3, among other things, is activated in response to IL-6 and IL-10 family of cytokines [31]. 

**Figure 5:**
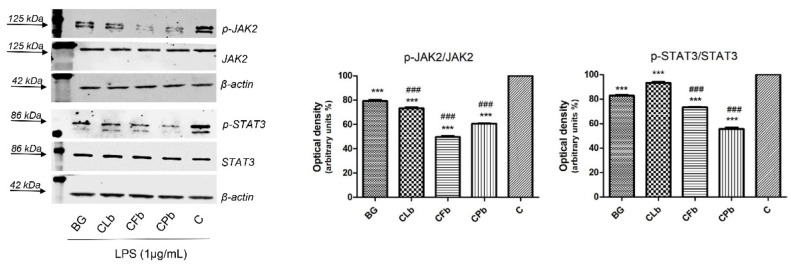
*Cachrys* extracts down-regulate p-JAK2 and p-STAT3 protein levels in RAW 264.7 macrophage cells. The expression of p-JAK2, total-JAK2, p-STAT3 and total-STAT3 was evaluated by Western blot analysis. Cells were lysed after 2 h as indicated in materials and methods. Equal amounts of total cellular extract (30 µg) were analyzed. β-Actin was used as loading control. The graphs show the densitometric analysis: phosphorylated forms were normalized on relative total proteins that have been previously normalized versus β-Actin. CLb: *C. libanotis*; CFb: *C. ferulacea*; CPb: *C. pungens*. Formulations were prepared as reported in the Materials and Methods Section 3.2. Significant difference versus control with LPS: *** *p* < 0.001; significant difference versus bergapten (BG, positive control): ### *p* < 0.001 (Dunnett’s multiple comparison test).

Starting from ELISA data and in perspective of Western blot analyses, one concentration from each investigated extract was selected for further analyses: for each sample, thanks to the good results coming from previous assay, the lowest one concentration was carried forward (CLb, CFb, CPb). Compared to bergapten, used as positive control, all the tested extracts significantly inhibited the phosphorylation of p-JAK2. As Figure 5 shows, CFb sample significantly reduced the phosphorylation of p-JAK2, followed by CPb. This last sample, instead, exerted the best inhibition of p-STAT3 phosphorylation, by significantly modulating activity if compared to bergapten. 

Even if to a minor extent, the CFb sample was also able to suppress p-STAT3 levels, while CLb resulted in an activity similar to bergapten. Correlating blot and ELISA analysis, it is clear that CPb and CFb exert the best activity in suppressing phosphorylate JAK and STAT proteins and in decreasing the release of pro-inflammatory cytokines (TNF-α, IL-6). CPb, specifically, induced the release of IL-10, an anti-inflammatory cytokine. Although the IL-6 cytokine activates a huge quantity of pathway, the IL-6 mediated STAT3 activation seems to be strictly correlated to tumorigenesis promotion processes [31]. 

The investigated extracts, whose content in furanocoumarins was standardized, were used in a such a dose that sum of furanocoumarins correspond to single dose of bergapten 10 µM, selected from literature. *Cachrys* extracts contain furanocoumarins, already known to be able to suppress the JAK/STAT signaling pathway. Zhou and coworkers [8], demonstrated that bergapten is effective in suppressing the release of pro-inflammatory cytokines and mediators in macrophages activated by LPS and, in the meantime, to inhibit the JAK/STAT signaling pathway. Xanthotoxin, another naturally occurring furanocoumarin described in *Cachrys* extract and quantified by GC-MS analysis (Table 2), significantly attenuates the pro-inflammatory cytokines production (TNF-α and IL-6) in RAW 264.7 cells stimulated with LPS and exerts anti-inflammatory activity by inhibiting the JAK/STAT pathway, as reported by Lee and colleagues [25]. Bergamottin, another furanocoumarin, even if not detected in the studied *Cachrys* extracts, inhibits constitutive STAT3 activation in multiple myeloma cells [30]. The coumarin imperatorin was demonstrated to inhibit the zymosan mediated upregulation of COX2, iNOS inflammatory protein and IL-6 and TNF-α inflammatory cytokines through a blockage of NF-kB and JAK1/STAT3 signaling pathway [32].

## 3. Materials and Methods

### 3.1. Reagents

Folin-Ciocalteu reagent, aluminum chloride, chlorogenic acid, quercetin, bergapten, fetal bovine serum (FBS), L-glutamine, penicillin/streptomycin, Dulbecco’s Modified Eagle’s Medium (DMEM), bovine serum albumin (BSA), protease inhibitors, trypan blue, phosphate buffered saline (PBS), LPS, trichloroacetic acid (TCA), sulphorhodamine B (SRB) were purchased by Sigma-Aldrich S.p.a. (Milan, Italy). ECL System was from Bio-Rad. RAW 264.7 cells were purchased by ATCC, UK (No. TIB-71). The Abs employed were anti-phosphoJak2 (#PA538287), anti-Jak2 (#PA511267), anti-phosphoStat3 (#PA5121259), anti-Stat3(#PA5120138), (all from ThermoFisher Scientific, Waltham, MA, USA), anti-β-actin (AC-15; sc-69879) from Santa Cruz Biotechnology, Inc., Heidelberg, Germany. All other reagents were obtained by VWR International s.r.l. (Milan, Italy).

### 3.2. Plant Material and Extraction Procedure

The whole epigeal part (flowers, stems and leaves) from *C. libanotis*, *C. ferulacea* and *C. pungens* were collected in Calabria, Southern Italy (leg. det. Carmine Lupia). Voucher specimens were deposited at Mediterranean Etnobotanical Conservatory, Sersale (Catanzaro), in Apiaceae section (numbers 18, 31 and 52, respectively). Dry plant material was cut into small pieces and extracted by Naviglio extractor^®^ (Atlas Filtri, s.r.l., Limena, PD, Italy) in methanol (MeOH) with the following conditions: plant:solvent ratio 1:10 g/mL, 2 cycles. Extracts were filtered using qualitative filter paper (particle retention 10–20 μm, VWR International, Leuven, Belgium) and dried at 37 °C under reduced pressure using a rotary evaporator (IKA^®^ RV 10, VWR International, Milan, Italy). Each extract was quantified and standardized in furanocoumarin content (xanthotoxin, bergapten and isopimpinellin) and two formulations were prepared for each extract: “a” and “b”, in which the sum of the three chosen standards was equal to the concentration of bergapten 10 µM (2.16 µg/mL) and 5 µM (1.08 µg/mL), two concentrations able to exert a biological activity without affecting cell viability. The concentration of bergapten 10 µM used as positive control was chosen based on previous literature studies [8] and tested in our conditions in order to confirm concentration and activity.

### 3.3. Determination of Total Phenolic and Flavonoid Content

Total phenolic content was assessed by means of Folin-Ciocalteu method described by Singleton and Rossi [33] and performed as reported by Rababah and colleagues [34]. 10 mg of extracts, previously dissolved in 5 mL of a mixture composed of acetone/methanol/water/acetic acid 40:40:20:0.1 were incubated at 60 °C for 1 h. Then, 200 µL of these solutions were added to 1 mL of Folin-Ciocalteu’s reagent and 1 mL of 7.5% sodium carbonate. Samples were incubated again, at room temperature for 2 h, then absorbance was measured at 726 nm with a UV/VIS spectrophotometer (Lambda 40, Perkin Elmer, Milan, Italy). Data were interpolated with a chlorogenic acid standard calibration curve and final results were expressed as mg of chlorogenic acid equivalents per g of dry plant material. 

Total flavonoid content was measured through the aluminum-chloride method. 10 mg of extracts were dissolved in 5 mL of EtOH 80% and then 1 mL of this mixture was added to 1 mL of AlCl_3_ reagent. After 15 min in the dark, absorbance was measured at 430 nm. Data were interpolated with quercetin standard calibration curve and final results were reported as mg of standard equivalent per g of dry plant material [35]. 

### 3.4. Qualitative and Quantitative Analyses Using Gas Chromatography—Mass Spectrometry (GC-MS)

Qualitative analyses were performed through a gas chromatograph (Hewlett-Packard 6890, Milan, Italy) equipped with a SE-30 (100% dimethylpolysiloxane) capillary column (30 m × 0.25 mm, 0.25 μm film thickness) coupled with a selective mass detector (Hewlett Packard 5973, Milan, Italy). A 10 µL GC microsyringe (Hamilton, Bonaduz, Switzerland) was used to inject samples (1 µL). Helium was used as carrier gas (linear velocity 0.00167 cm/s). Analyses were performed with a programmed temperature scheme that provides an increasing temperature from 60 °C to 280 °C and a rate of 16°/min (Figure S2). Column inlet was set at 250 °C. Operating parameters were as follows: ion source, 70 eV; ion source temperature, 230 °C; electron current, 34.6 μA; vacuum 10^−5^ torr. Mass spectra were acquired over a 40–800 amu range at 1 scan/s. Compounds were identified by comparing them with mass spectra contained in the Wiley 138 library data. 

A quantitative analysis was also performed in order to assess the specific amount of three furanocoumarins in *Cachrys* extracts: xanthotoxin, bergapten and isopimpinellin. Bergapten (10–0.16 mg/mL in MeOH/CHCl_3_) was used as external standard: a linear regression equation y = 1E + 07x + 2E + 06; R^2^ = 0.9949 was created. Analyses were carried out in isothermal conditions at 235 °C, and peaks area were evaluated in triplicate as previously reported [11].

### 3.5. Cell Cultures

RAW 264.7 macrophages were purchased from ATCC, UK (No. TIB-71), and used within 4 months after frozen aliquots resuscitations (less than 30 passages). Cells were tested monthly for negativity to mycoplasma using the MycoAlert Mycoplasma Detection Kit (Lonza, Gampel-Bratsch, Switzerland). Cells were cultured in Dulbecco’s Modified Eagle’s Medium (DMEM) implemented with 10% fetal bovine serum (FBS), 1% L-glutamine and 1% penicillin/streptomycin and incubated at 37 °C in 5% CO_2_ atmosphere.

### 3.6. Cell Viability Assay (SRB)

The sulphorhodamine B (SRB) assay was used to evaluate cell viability by staining cellular protein content. 3000 cells/well were seeded into a 96-well plate and incubated to allow attachment. After 24 h, cells were pre-treated with “a” and “b” formulations of each *Cachrys* extracts and with bergapten (10 µM), and stimulated with LPS (1 µg/mL) 30 min later. After further 24 h, treated cells were fixed with 10% trichloroacetic acid (TCA) for 1 h at 4 °C. Then, SRB was added for staining and cells were washed 3 times with a 1% acetic acid solution. Absorbance was measured at 540 nm using a microplate reader (Stat fax 3200, Awareness Technology Inc., Palm City, FL, USA). [36].

### 3.7. Western Blot Analysis

Cells were treated with samples and LPS and they were lysed in RIPA lysis buffer [20 mM Tris-HCl (pH 7.5), 150 mM NaCl, 1 mM Na2 EDTA, 1 mM EGTA, 1% NP-40, 1% sodium deoxycholate, 2.5 mM sodium pyrophosphate] supplemented with protease inhibitor cocktail and phosphatase inhibitor two hours later. Equal amounts of protein extracts (30 µg) were subjected to SDS-PAGE gel 11% and then electroblotted onto a nitrocellulose membrane, as previously described [37]. Proteins were detected with specific polyclonal (p) or monoclonal (m) antibodies (Abs), as indicate in Section 3.1, and recognized by IRDye secondary Abs (LI-COR Corporate, Milan, Italy). Western blot images are representative of at least three independent experiments and acquired with the Odyssey FC Imaging System (LI-COR Corporate). For each Western blot figure, the whole blots showing all the bands with all molecular weight markers, as well as the densitometry readings/intensity ratio of each band were analyzed using Image J software (NIH, Bethesda, MD, USA).

### 3.8. Cytokine Measurements

Cells were seeded into 24-well plates (3 × 10^5^ cells/well), incubated for 24 h, treated and activated as previously described. After 24 h, media was collected and TNF-α, IL-6 and IL-10 were evaluated by ELISA kits (ThermoFisher Scientific, Bender MedSystems GmbH, Vienna, Austria) according to the manufacturer’s instructions.

### 3.9. Nitrite Analysis

Cells were seeded into 24-well plates (3 × 10^5^ cells/well) and incubated for 1 day. 24 h after treatments and LPS stimulation, media was collected and mixed with the same volume of Griess reagent, used to evaluate the presence of nitrite, stable oxidized NO products. Absorbance was measured at 550 nm using a UV/VIS spectrophotometer (Stat fax 3200, Awareness Technology Inc., Palm City, FL, USA). [38].

### 3.10. Statistical Analysis

Experiments were run in triplicates (n = 3), except for SRB test, which was performed in quadruplicate (n = 4). Data are expressed as mean ± S.E.M. Normality of data was evaluated through D’Agostino-Pearson’s K2 test; homogeneity of variances was estimated by Levene’s test. Statistical differences between treated groups and the control (Dunnett’s multiple comparison test), among treated group means (Bonferroni post-hoc test) and among two samples (Student’s *t* test) were assessed through one-way ANOVA (Graph-Pad Prism Software 5, San Diego, CA, USA and SigmaStat Software SanRafael, CA, USA). Western blot analyses were analyzed on Image J software (NIH, Bethesda, MD, USA). Principal component analysis (PCA) was carried out using the MetaboAnalyst software v. 5.0 (http://www.metaboanalyst.ca (accessed on 28 July 2022)). Data were checked for integrity. Zero values were replaced with small values (1/5 of the min positive value for each variable found within the original data), and data were pretreated through Pareto-scaling. The chemical constituents identified in the three *Cachrys* species were graphically represented through clustered heat mapping technique.

## 4. Conclusions

As reported above, several different evidences showed that coumarins exert anti-inflammatory activity via the JAK/STAT signaling pathway [39]. Different studies focusing on the phytochemical composition of *Cachrys* species reported they are abundant in coumarins and their derivatives, mainly furanocoumarins [40,41,42,43]. Starting from this consideration, aim of this work was to investigate three species present in Southern Italy. *Cachrys pungens* extract, standardized in furanocoumarin content (xanthotoxin, bergapten and isopimpinellin), was significantly able to inhibit the phosphorylation of STAT3 by suppressing the release of pro-inflammatory cytokines (TNF-α, IL-6) and by increasing levels of IL-10 anti-inflammatory cytokine, while *Cachrys ferulacea* was particularly effective in decreasing the p-JAK2 protein levels in LPS-stimulated RAW 264.7 cells. In conclusion, this study showed that *Cachrys* extracts could be considered a potential source of anti-inflammatory agents able to downregulate the JAK/STAT signaling pathway.

## Figures and Tables

**Table 1:** Determination of yield, total phenolic and flavonoid content of investigated *Cachrys* species.

Species	TP ^1^	TF ^2^	Yield (%)
*C. libanotis* L. ^3^	12.80 ± 0.10 ^a^	0.09 ± 0.01 ^c^	12.6
*C. ferulacea* (L.) Calest.	4.14 ± 0.24 ^c^	0.18 ± 0.02 ^b^	3.6
*C. pungens* Jan	8.64 ± 0.42 ^b^	0.32 ± 0.01 ^a^	7.4

^1^ Total Phenolic content. ^2^ Total Flavonoid content. Data are expressed as mean ± S.D. (n = 3). ^3^ Marrelli et al., 2021 [11]. Results are expressed as mg of chlorogenic acid (for phenolics) and mg of quercetin (for flavonoids) per g of dry plant material. Different letters along columns indicate statistical differences at *p* < 0.05 (Bonferroni post-hoc test).

**Table 2:** Quantitative analysis of identified furanocoumarins.

Species	Xanthotoxin	Bergapten	Isopimpinellin
*C. libanotis* L. ^1^	4.98 ± 0.21	0.59 ± 0.08	0.42 ± 0.03
*C. ferulacea* (L.) Calest.	0.42 ± 0.05	0.57 ± 0.04	0.050 ± 0.001
*C. pungens* Jan	7.48 ± 0.48	2.94 ± 0.16	1.07 ± 0.14

Data are expressed as mean ± S. D. (n = 3). Statistical differences are shown in Figure 2. ^1^ Marrelli et al., 2021 [11].

**Table 3:** Investigated *Cachrys* species and extract formulations “a” and “b”, prepared as reported in the Materials and Methods Section 3.2.

Sample	Extract Formulation	Abbreviation
*C. libanotis* L.	a	CLa
	b	CLb
*C. ferulacea* (L.) Calest.	a	CFa
	b	CFb
*C. pungens* Jan	a	CPa
	b	CPb

## Data Availability

The data presented in this study are available in the article.

## Supplementary Materials

The following supporting information can be downloaded at: https://www.mdpi.com/article/10.3390/plants11212913/s1, Figure S1: (a) *C. libanotis* L., (b) *C. ferulacea* (L.) Calest., (c) *C. pungens* Jan.; Figure S2: GC-MS chromatograms of (a) *C. libanotis* L., (b) *C. ferulacea* (L.) Calest. and (c) *C. pungens* Jan extracts.

## References

[1] Les F., Cásedas G., López V. (2021). Bioactivity of medicinal plants and extracts. Biology.

[2] Tiwari R., Rana C.S. (2015). Plant secondary metabolites: A review. IJERGS.

[3] Ryabushkina N.A. (2005). Synergism of metabolite action in plant responses to stresses. Russ. J. Plant Physiol..

[4] Morris R., Kershaw N.J., Babon J.J. (2018). The molecular details of cytokine signaling via the JAK/STAT pathway. Protein Sci..

[5] Zobel A.M., Wang J., March R.E., Brown S.A. (1991). Identification of eight coumarins occurring with psoralen, xanthotoxin, and bergapten on leaf surfaces. J. Chem. Ecol..

[6] Quetglas-Llabrés M.M., Quispe C., Herrera-Bravo J., Catarino M.D., Pereira O.R., Cardoso S.M., Dua K., Chellappan D.K., Pabreja K., Satija S. (2022). Pharmacological properties of bergapten: Mechanistic and therapeutic aspects. Oxid. Med. Cell Longev..

[7] Liang Y., Xie L., Liu K., Cao Y., Dai X., Wang X., Lu J., Zhang X., Li X. (2021). Bergapten: A review of its pharmacology, pharmacokinetics, and toxicity. Phytother. Res..

[8] Zhou Y., Wang J., Yang W., Qi X., Lan L., Luo L., Yin Z. (2017). Bergapten prevents lipopolysaccharide-induced inflammation in RAW264. 7 cells through suppressing JAK/STAT activation and ROS production and increases the survival rate of mice after LPS challenge. Int. Immunopharmacol..

[9] Denaro M., Smeriglio A., Xiao J., Cornara L., Burlando B., Trombetta D. (2020). New insights into Citrus genus: From ancient fruits to new hybrids. Food Front..

[10] Bruno M., Ilardi V., Lupidi G., Quassinti L., Bramucci M., Fiorini D., Venditti A., Maggi F. (2019). The nonvolatile and volatile metabolites of Prangos ferulacea and their biological properties. Planta Med..

[11] Marrelli M., Perri M.R., Amodeo V., Giordano F., Statti G.A., Panno M.L., Conforti F. (2021). Assessment of photo-Induced cytotoxic activity of *Cachrys sicula* and *Cachrys libanotis* enriched-Coumarin extracts against human melanoma cells. Plants.

[12] Ahmed S., Khan H., Aschner M., Mirzae H., Küpeli A.E., Capasso R. (2020). Anticancer potential of furanocoumarins: Mechanistic and therapeutic aspects. Int. J. Mol. Sci..

[13] Martin Y.C., Kofron J.L., Traphagen L.M. (2002). Do structurally similar molecules have similar biological activity?. J. Med. Chem..

[14] Willett P. (2006). Similarity-Based virtual screening using 2D fingerprints. Drug Discov. Today.

[15] Naviglio D. (2003). Naviglio’s principle and presentation of an innovative solid–Liquid extraction technology: Extractor Naviglio^®^. Anal. Lett..

[16] Naviglio D., Pizzolongo F., Romano R., Ferrara L., Naviglio B., Santini A. (2007). An innovative solid-Liquid extraction technology: Use of the Naviglio extractor^®^ for the production of lemon liquor. Afr. J. Food Sci..

[17] Klepacka J., Gujska E., Michalak J. (2011). Phenolic compounds as cultivar-and variety-Distinguishing factors in some plant products. Plant Foods Hum. Nutr..

[18] Eseberri I., Trepiana J., Léniz A., Gómez-García I., Carr-Ugarte H., González M., Portillo M.P. (2022). Variability in the Beneficial Effects of Phenolic Compounds: A Review. Nutrients.

[19] Boneza M.M., Niemeyer E.D. (2018). Cultivar affects the phenolic composition and antioxidant properties of commercially available lemon balm (*Melissa officinalis* L.) varieties. Ind. Crops Prod..

[20] Amin I., Norazaidah Y., Hainida K.E. (2006). Antioxidant activity and phenolic content of raw and blanched *Amaranthus* species. Food Chem..

[21] Jeshvaghani Z.A., Rahimmalek M., Talebi M., Goli S.A.H. (2015). Comparison of total phenolic content and antioxidant activity in different Salvia species using three model systems. Ind. Crops Prod..

[22] Nickavar B., Esbati N. (2012). Evaluation of the antioxidant capacity and phenolic content of three *Thymus* species. J. Acupunct. Meridian Stud..

[23] Bazdar M., Sadeghi H., Hosseini S. (2018). Evaluation of oil profiles, total phenols and phenolic compounds in Prangos ferulacea leaves and flowers and their effects on antioxidant activities. Biocatal. Agric. Biotechnol..

[24] Marrelli M., Perri M.R., Amodeo V., Conforti F., Giordano F., Panno M.L., Statti G. (2021). *Cachrys ferulacea* (L.) Calest. Extracts as Natural Photosensitizers: An In Vitro Photobiological Study. Biol. Life Sci. Forum.

[25] Lee S.B., Lee W.S., Shin J.S., Jang D.S., Lee K.T. (2017). Xanthotoxin suppresses LPS-induced expression of iNOS, COX-2, TNF-α, and IL-6 via AP-1, NF-κB, and JAK-STAT inactivation in RAW 264.7 macrophages. Int. Immunopharmacol..

[26] Bose S.K., Dewanjee S., Sahu R., Dey S.P. (2011). Effect of bergapten from *Heracleum nepalense* root on production of proinflammatory cytokines. Nat. Prod. Res..

[27] Singh G., Kaur A., Kaur J., Bhatti M.S., Singh P., Bhatti R. (2019). Bergapten inhibits chemically induced nociceptive behavior and inflammation in mice by decreasing the expression of spinal PARP, iNOS, COX-2 and inflammatory cytokines. Inflammopharmacology.

[28] Yang Y., Zheng K., Mei W., Wang Y., Yu C., Yu B., Deng S., Hu J. (2018). Anti-Inflammatory and proresolution activities of bergapten isolated from the roots of *Ficus hirta* in an in vivo zebrafish model. Biochem. Biophys. Res. Commun..

[29] Wang C.C., Lai J.E., Chen L.G., Yen K.Y., Yang L.L. (2000). Inducible nitric oxide synthase inhibitors of Chinese herbs. Part 2: Naturally occurring furanocoumarins. Bioorg. Med. Chem..

[30] Kim S.M., Lee J.H., Sethi G., Kim C., Baek S.H., Nam D., Chung W.S., Kim S.H., Shim B.S., Ahn K.S. (2014). Bergamottin, a natural furanocoumarin obtained from grapefruit juice induces chemosensitization and apoptosis through the inhibition of STAT3 signaling pathway in tumor cells. Cancer Lett..

[31] Sansone P., Bromberg J. (2012). Targeting the interleukin-6/Jak/stat pathway in human malignancies. J. Clin. Oncol..

[32] Li Y.Z., Chen J.H., Tsai C.F., Yeh W.L. (2019). Anti-Inflammatory property of imperatorin on alveolar macrophages and inflammatory lung injury. J. Nat. Prod..

[33] Singleton V.L., Rossi J.A. (1965). Colorimetry of total phenolics with phosphomolybdic-Phosphotungstic acid reagents. Am. J. Enol. Vitic..

[34] Rababah T.M., Hettiarachchy N.S., Horax R. (2004). Total phenolics and antioxidant activities of fenugreek, green tea, black tea, grape seed, ginger, rosemary, gotu kola, and ginkgo extracts, vitamin E, and tert-butylhydroquinone. J. Agric. Food Chem..

[35] Djeridane A., Yousfi M., Nadjemi B., Boutassouna D., Stocker P., Vidal N. (2006). Antioxidant activity of some Algerian medicinal plants extracts containing phenolic compounds. Food Chem..

[36] Mazzei R., Piacentini E., Nardi M., Poerio T., Bazzarelli F., Procopio A., Di Gioia M.L., Rizza P., Ceraldi R., Morelli C. (2020). Production of plant-derived oleuropein aglycone by a combined membrane process and evaluation of its breast anticancer properties. Front. Bioeng. Biotechnol..

[37] Rizza P., Barone I., Zito D., Giordano F., Lanzino M., De Amicis F., Mauro L., Sisci D., Catalano S., Wright K.D. (2014). Estrogen receptor beta as a novel target of androgen receptor action in breast cancer cell lines. Breast Cancer Res..

[38] Marrelli M., De Marco C.T., Statti G., Neag T.A., Toma C.C., Conforti F. (2022). *Ranunculus* species suppress nitric oxide production in LPS-Stimulated RAW 264.7 macrophages. Nat. Prod. Res..

[39] Bose S., Banerjee S., Mondal A., Chakraborty U., Pumarol J., Croley C.R., Bishayee A. (2020). Targeting the JAK/STAT signaling pathway using phytocompounds for cancer prevention and therapy. Cells.

[40] Abad M.J., De las Heras B., Silvan A.M., Pascual R., Bermejo P., Rodriguez B., Villar A.M. (2001). Effects of furocoumarins from *Cachrys trifida* on some macrophage functions. J. Pharm. Pharmacol..

[41] Grande M., Aguado M.T., Mancheño B., Piera F. (1986). Coumarins and ferulol esters from *Cachrys sicula*. Phytochemistry.

[42] Pistelli L., Catalano S., Manunta A., Marsili A. (1989). Coumarins from *Cachrys ferulacea* collected in Sardinia. Planta Med..

[43] Tahar S., Hamdi B., Flamini G., Mehmet Ö., Duru M.E., Bruno M., Maggi F. (2022). Chemical composition, antioxidant and anticholinesterase activity of the essential oil of algerian *Cachrys sicula* L.. Nat. Prod. Res..

