# Supplementary material for: Cachrys spp. from Southern Italy: Phytochemical Characterization and JAK/STAT Signaling Pathway Inhibition
Source: Plants (Basel). 2022 Oct 29;11(21):2913. doi: 10.3390/plants11212913 (PMC9655331; doi:10.3390/plants11212913)
Supplement: Supplementary file 1 [file plants-11-02913-s001.zip › plants-1980396-supplementary.pdf]

(a) 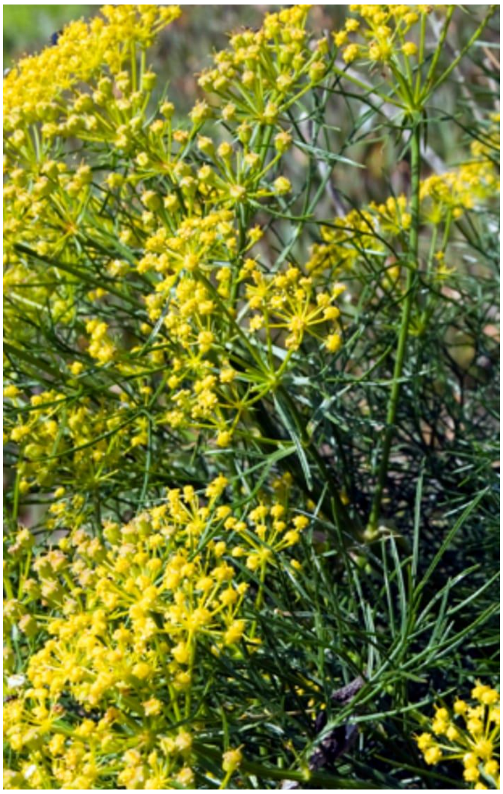

(b) 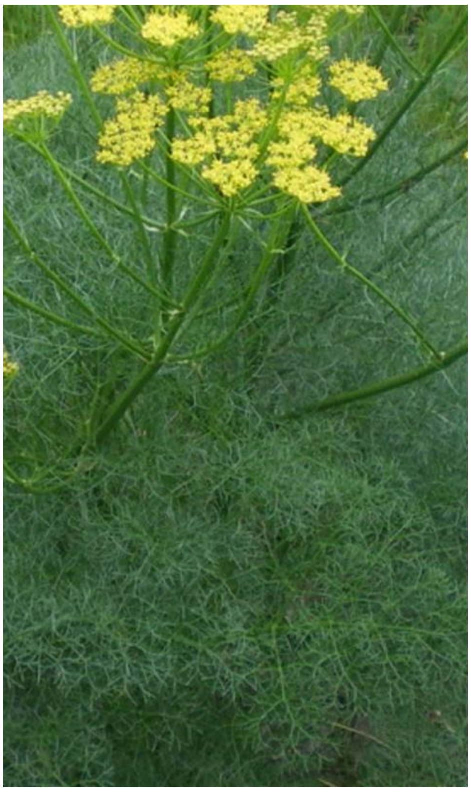

(c) 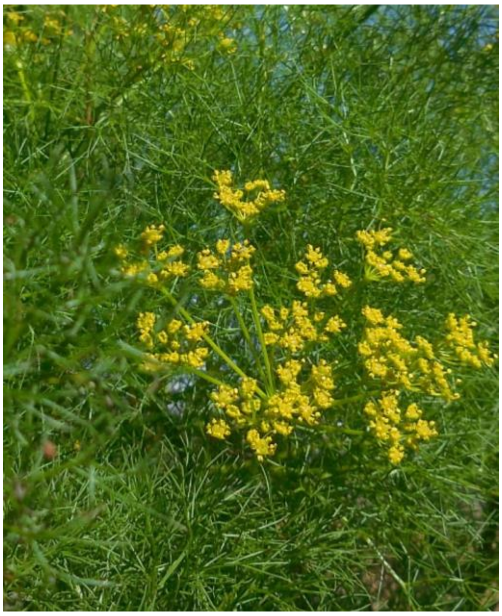

Figure S1. (**a**) *C. libanotis* L., (**b**) *C. ferulacea* (L.) Calest., (**c**) *C. pungens* Jan.

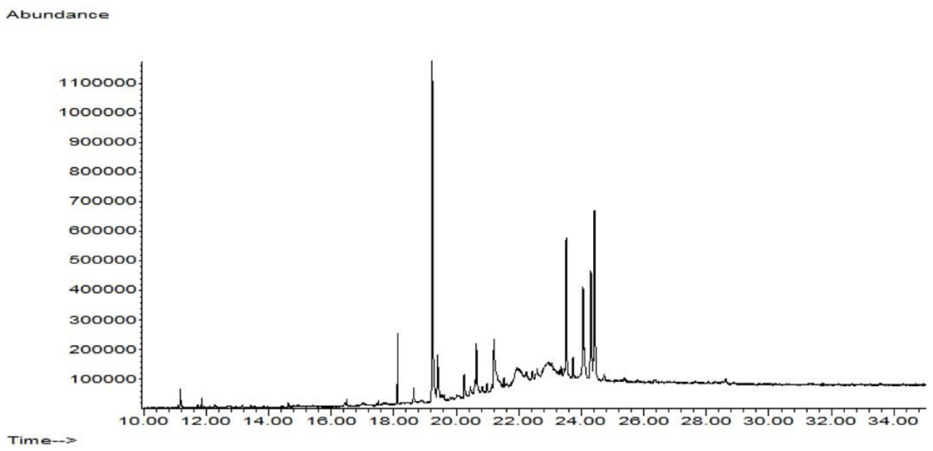

(**a**)

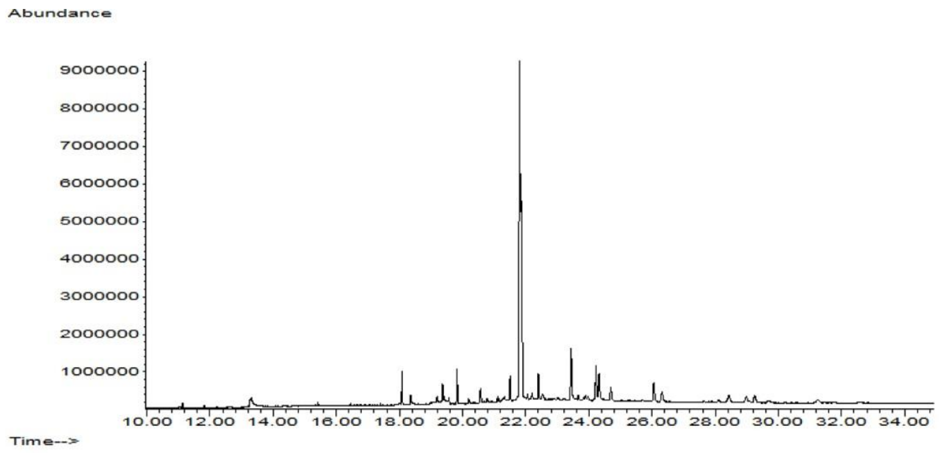

(**b**)

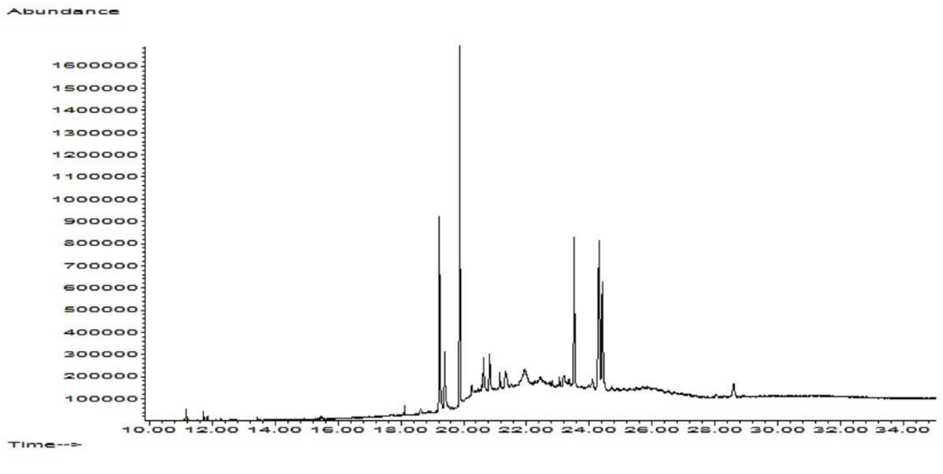

(**c**)

Figure S2. GC-MS chromatograms of (**a**) *C. libanotis* L., (**b**) *C. ferulacea* (L.) Calest., (**c**) *C. pungens* Jan extracts.
